# Ethnopharmacology, Chemistry and Biological Properties of Four Malian Medicinal Plants

**DOI:** 10.3390/plants6010011

**Published:** 2017-02-21

**Authors:** Karl Egil Malterud

**Affiliations:** Section Pharmacognosy, Department of Pharmaceutical Chemistry, School of Pharmacy, University of Oslo, P.O. Box 1068 Blindern, Oslo 0316, Norway; k.e.malterud@farmasi.uio.no; Tel.: +47-22-856563

**Keywords:** Malian medicinal plants, *Biophytum umbraculum*, *Burkea africana*, *Lannea velutina*, *Terminalia macroptera*

## Abstract

The ethnopharmacology, chemistry and pharmacology of four Malian medicinal plants, *Biophytum umbraculum*, *Burkea africana*, *Lannea velutina* and *Terminalia macroptera* are reviewed. These plants are used by traditional healers against numerous ailments: malaria, gastrointestinal diseases, wounds, sexually transmitted diseases, insect bites and snake bites, etc. The scientific evidence for these uses is, however, limited. From the chemical and pharmacological evidence presented here, it seems possible that the use in traditional medicine of these plants may have a rational basis, although more clinical studies are needed.

## 1. Introduction

Africa has a very varied flora, and the study of African medicinal plants has engaged many scientists for a long time. The oldest written documents on this are found in the Papyrus Ebers, written ca. 3500 years ago [1,2]. One early study by the Norwegian medical doctor Henrik Greve Blessing, carried out in 1901–1904, but only existing as a handwritten manuscript, was recently discovered and has now been published [3].

Studies on African medicinal plants have in nearly all cases been limited to geographically limited areas—this is necessary, due to the very wide floral variation throughout this huge continent. Some examples of the large number of books dealing with African medicinal plants are the classical work by Watt and Breyer-Brandwijk [4] and Burkill’s multivolume treatise [5]. A few more recent examples include the books by Iwu [1], Kuete [6], Neuwinger [7] and van Wyk [8]—this list is very far from complete! Journals such as *African Journal of Traditional, Complementary and Alternative Medicines*, *Journal of Ethnopharmacology*, *Journal of Ethnobiology and Ethnomedicine*, as well as many others, are also rich sources of knowledge of African medicinal plants and their properties and use.

For many years, scientists in Mali and Norway have collaborated in investigating Malian medicinal plants, and this has resulted in more than sixty publications in peer-reviewed international journals, numerous contributions at national and international conferences, nine Ph.D. degrees and more than 100 M.Sc. degrees awarded. A description of this project is available on the web [9].

In this brief survey, I intend to review the ethnopharmacology, chemistry and pharmacology of some Malian medicinal plants. The plants discussed have been studied in my research group in the Section of Pharmacognosy, School of Pharmacy, University of Oslo. Our research has in the main been directed towards isolation and identification of secondary metabolites of plant origin and their properties as antioxidants, radical scavengers and inhibitors of enzymes involved in peroxidative processes.

Four of the less known species are chosen for this treatise, viz. *Biophytum umbraculum*, *Burkea africana*, *Lannea velutina* and *Terminalia macroptera* (Table 1). References to previous work were found through the SciFinder database, which covers Medline and Chemical Abstracts. Titles in other languages than English, French or German have been used in translation.

A recent review [15] covers medicinal plants from Mali. That review is, however, differently angled, and the plants covered in detail in the present article are only briefly mentioned.

## 2. *Biophytum umbraculum*

*Biophytum umbraculum* Welw. (Oxalidaceae) is a small herb, up to 15 cm in height. It is widespread in the tropical parts of Africa and Asia [16]. Several synonyms exist for this plant, including *B. petersianum* Klotzsch (the name most commonly found in the scientific literature) [17].

### 2.1. Ethnopharmacology

Few systematic surveys on the traditional use of this plant have been done. Diallo et al. [18] reported that leaves are used in wound healing, using the dried and powdered plant. Another survey [19] in Mali found that while the plant was used against pain, insect bites and snake bites, treatment of cerebral malaria was by far the most common indication.

### 2.2. Chemistry

The polysaccharides of this plant are fairly well investigated. Pectic polysaccharides with several biological activities have been isolated and identified [20,21,22]. These polysaccharides contain rhamnogalacturonan, xylogalacturonan, and arabinogalactan regions. Very little was known about the low molecular weight constituents prior to our studies. Saponins were known to be present in the plant [23,24], but their structures still remain unknown.

**Figure 1:**
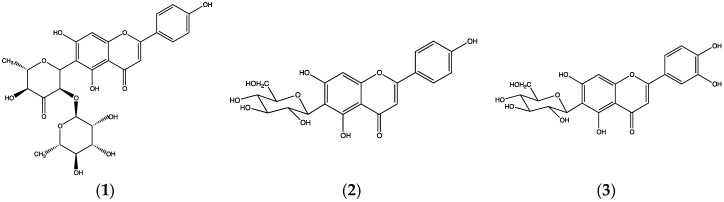
Cassiaoccidentalin A (**1**); isovitexin (**2**) and isoorientin (**3**) from *B. umbraculum.*

From a methanol extract of above ground parts of *B. umbraculum*, we isolated the *C*-glycosylflavones cassiaoccidentalin A (**1**), isovitexin (**2**) and isoorientin (**3**) (Figure 1) [10]. Isovitexin and isoorientin are not rare substances, but this appears to the first report on their occurrence in *B. umbraculum*. They have, however, been reported from another *Biophytum* species, *B. sensitivum* [25].

Cassiaoccidentalin A was first reported from *Cassia occidentalis* (Leguminosae) [26] along with substances (**2**) and (**3**). After our report on this, cassiaoccidentalin A was found in *Serjania marginata* (Sapindaceae) leaves [27]. To our knowledge, no other sources for this compound are known.

### 2.3. Biological Activity

Crude extracts of the plant show complement fixing activity [18], hypotensive effects [28], calcium antagonism [29], and increased corticosteroid secretion [30]. Decreased methane production was observed in cattle fed *B. petersianum* [24].

The polysaccharides referred to in Section 2.2 have been investigated for immunological effects. Complement fixing, macrophage activation, dendritic cell activation and immunomodulating activity against Peyer’s patch cells has been reported [20,21,22]. The pectic polysaccharides from this plant exhibited protective ability against *Streptococcus pneumoniae* in a mouse model [31]. Cassiaoccidentalin A was investigated for suppression of the HIV promoter [32], but was found to be without effect. The plant where it was first found, *C. occidentalis*, is a known antimalarial plant and is used in Mali as one of the components of the “Improved Traditional Medicine” Malarial [33], which has been subjected to clinical studies. In view of the traditional use in Mali against cerebral malaria and the related chemistry between *B. umbraculum* and *C. occidentalis*, we investigated the malaria-related and antiinflammatory properties of *B. umbraculum* extracts and pure substances [34]. The ethyl acetate extract showed antiplasmodial, anti-complement and antiinflammatory activity and inhibited lipopolysaccharide stimulation in macrophages, but this appears to be due to other constituents than the isolated flavonoids. These findings may be relevant for the ethnopharmacological use of the plant against cerebral malaria. No clinical studies have been done, however.

## 3. *Burkea africana*

The tree *Burkea africana* Hook. (Leguminosae), usually less than 10 m in height, grows over large parts of tropical and subtropical Africa [35]. No synonyms are given for this plant [17].

### 3.1. Ethnopharmacology

In traditional medicine, the powdered stem bark is used in Mali on wounds in the mouth [18]. Other sources [4,35,36,37,38] cite a number of uses in different African countries.

### 3.2. Chemistry

Like many African plants, *B. africana* has only been subjected to a limited number of scientific studies. It has been reported [39] that the activity of the bark, which was stated as being used in folk medicine against gastrointestinal symptoms and headache, was due to its content of tyramine. The finding of tyramine was later corroborated [40], and the occurrence of harman-type alkaloids was reported, as well [41,42]. 5-Hydroxypipecolic acid, an unusual amino acid, was reported from the seeds of this plant [43].

Work in Ferreira’s group in the late 1980s [44,45] led to the identification of several new oligomeric flavonoids (proanthocyanidins) based on the flavan-3-ol fisetinidol (5-deoxycatechin) in the heartwood of *B. africana*. The leaf lipids [46] and the stem bark polysaccharides [18] have been investigated, as well.

In view of the widespread medicinal use and the limited knowledge of the constituents of this plant, we decided to investigate the chemistry and the antioxidative properties of the stem bark, which appears to be the part of the plant that is most commonly used for medicinal purposes in Mali. The putative wound-healing and antiinflammatory activities ascribed to preparations of *B. africana* stem bark might well be correlated to antioxidative effects.

**Figure 2:**
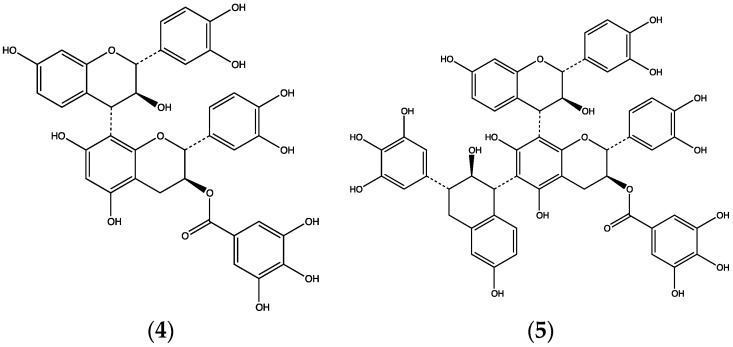
Structures of compounds (**4**) and (**5**) from *Burkea africana* stem bark.

Using stem bark collected in Blendio, about 320 km south-east of the capital Bamako, we made an ethanolic extract which was subjected to liquid-liquid partition and repeated chromatography. This led to two major constituents (Figure 2), one of which was identified as fisetinidol-(4β→8)-catechin-3-*O*-gallate (**4**), previously known only from the heartwood of this tree [45]. The other substance was given the structure bis-fisetinidol-(4→6, 4→8)-catechin-3-*O*-gallate (**5**). Compound (**5**) was a new natural product [11].

### 3.3. Biological Activity

A pharmacological screening of Malian medicinal plants [47] revealed that a methanolic bark extract of *B. africana* had considerable radical scavenging activity as well as molluscicidal properties, and that both dichloromethane and methanol extracts of the bark were fungicidal. The methanolic bark extract has later been shown to attenuate oxidative stress in cells [48]. Fungicidal effects have been reported for heartwood extracts, as well [49], and moderate activity of water and methanol extracts of the bark against *Candida albicans* was reported [50]. An ethanol extract of the stem bark was shown to be antidiarrhoeal in mice [51]. Moderate inhibition of hyaluronidase and phospholipase A_2_ as well as antiproteolytic activity of methanol and water extracts of *B. africana* was reported [52]. An acetone leaf extract was tested for some antioxidant and antiinflammatory activities, finding inhibition of 15-lipoxygenase, acetylcholinesterase and nitric oxide production, as well as radical scavenging activity in different assay systems [53].

We found [11,54] that the crude aqueous ethanolic extract (80% ethanol) had very high radical scavenging activity, good 15-lipoxygenase inhibitory activity, and was also active as an inhibitor of iron-induced peroxidation of phospholipids. Of the subfractions from liquid-liquid partition, the ethyl acetate part was the most active, indicating that the active constituents were semipolar.

Substances **4** and **5** were about equiactive as radical scavengers and 15-lipoxygenase inhibitors, as might be expected from their polyphenolic structures, but differed in their inhibitory activity against phospholipid peroxidation.

We concluded that the hydroethanolic extract of *Burkea africana* stem bark showed strong activity against several peroxidative processes, probably due to its high content of polyphenolic substances, most of which appear to be proanthocyanidin-type tannins [11,54]. Wound-healing properties of tannins are well described in the literature (e.g., [55,56]), so our findings may be related to the ethnopharmacological use of the plant. Although animal experiments have been reported, it appears that no clinical studies have been carried out.

## 4. *Lannea velutina*

*Lannea velutina* A. Rich. (Anacardiaceae), syn. *Calesiam velutinum* (A. Rich.) Kuntze, *Odina velutina* (A. Rich.) Oliv. [17] is a shrub or tree growing from Senegal to Ghana.

### 4.1. Ethnopharmacology

Several sources report on the medicinal use of this plant, but without details [57,58]. More specifically, decoctions are said to be used against diarrhoea, rachitis, muscle pains, gastric pains, and as a tonic. The bark is applied to wounds and leprosy [59]. In Mali, the stem bark is used in wound healing [18], and the leaves are sold in markets for making a decoction used as an antidote to poisoning [60]. The fruits are edible [61]. According to one source, the smoke from the twigs is used in witchcraft [62]. In our field work, we found that in Dioila and Kolokani, leaf decoctions, stem bark decoctions and root decoctions of *L. velutina* are used against a multitude of diseases, including skin diseases, fever, gastrointestinal diseases, and in wound treatment [12,63].

### 4.2. Chemistry

**Figure 3:**
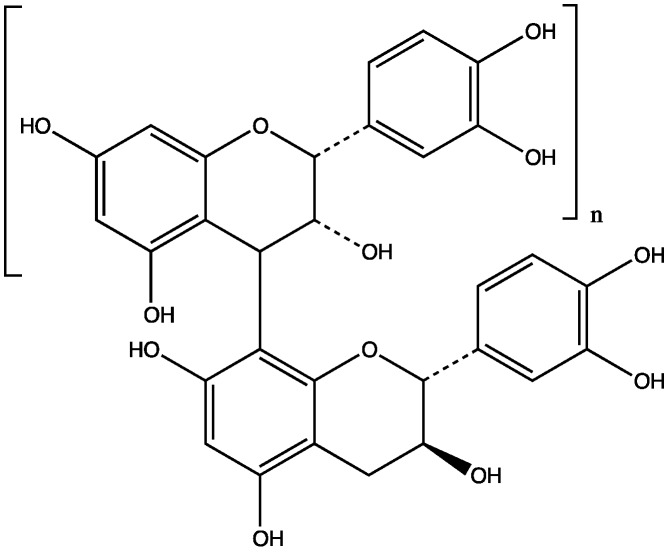
General structure of procyanidins from *L. velutina* (lower part: catechin, upper part: epicatechin, *n* = 0 (catechin monomer) to 11 (procyanidin dodecamer).

Prior to our studies, this plant appeared to be nearly uninvestigated. Unidentified tannins are reported to occur in all parts of the plant, mainly in the bark [64]. We found that the root bark of this plant was a rich source of procyanidins [63]. These had a linear 4→8 structure and commonly had catechin as starter unit and epicatechin as extender unit. Monomeric (catechin), dimeric, trimeric, tetrameric, hexameric, heptameric, nonameric, decameric and dodecameric procyanidin fractions were isolated (Figure 3). Most of the procyanidins were decameric or higher.

Mass spectroscopy revealed that *O*-methylation, *O*-galloylation and substitution of epiafzelechin (deoxyepicatechin) for epicatechin occurred, although to a minor extent. 

### 4.3. Biological Activity

In a survey of Malian medicinal plants [47], extracts (dichloromethane, methanol, water) of the leaves, bark and roots of *L. velutina* were investigated for antifungal, larvicidal, molluscicidal, antioxidant and radical scavenging activities. Although activities varied between plant parts and extracts, positive results were obtained for antioxidant activity (bark and root methanol extracts), antifungal activity (leaf dichloromethane extract active against both *Candida albicans* and *Cladosporium cucumerinum*, the other extracts had more selective activity), larvicidal activity against the malaria mosquito *Anopheles gambiae* (dichloromethane bark and leaf extract, methanol leaf extract), and molluscicidal activity towards the schistomiasis-transmitting snail *Biomphalaria pfeifferi*. Antioxidant and antibacterial activity of an ethanol extract of *L. velutina* bark were reported [65], with the highest activity towards *Bacillus subtilis, Staphylococcus aureus* (Gram-positive), *Pseudomonas aeruginosa* and *Salmonella typhimurium* (Gram-negative).

We investigated the radical scavenger and 15-lipoxygenase inhibitory activity of different extracts of root bark and stem bark [12]. Lipophilic extracts (petrol ether, dichloromethane, chloroform) were inactive as scavengers of the DPPH radical, water extracts had moderate activity, while semipolar extracts (methanol, 80% aqueous ethanol) of both root bark and stem bark were highly active. Similar observations (high activity of semipolar extracts) were made for inhibition of the peroxidative enzyme 15-lipoxygenase, although lipophilic extracts were more active than aqueous extracts in this assay. In a continuation of these experiments [63], radical scavenging activity and 15-lipoxygenase inhibition were investigated for the purified proanthocyanidins. All of them were highly active, although there was a slight trend towards higher activity on a weight basis for the higher molecular weight substances in both assays, translating to a clear molecular weight—activity correlation on a molecular basis. Two of the smaller compounds, epicatechin and trimeric proanthocyanidin (mostly catechin-→epicatechin→epicatechin) were tested for ability to counteract pro-oxidant toxicity induced by glutamate in cerebellar neurons. The trimer gave a significant decrease in cell death. Although a decrease was observed for epicatechin, as well, this did not reach statistical significance.

As mentioned above (Section 3.3), the contents of proanthocyanidins may be of relevance to the ethnopharmacological use of the plants against wounds. Proanthocyanidins may also be involved in the use of the plant against gastric pains and diarrhea [66]. As for many ethnopharmacologically used plants, clinical data are lacking.

## 5. *Terminalia macroptera*

*Terminalia macroptera* Guill. & Perr. (Combretaceae) (syn. *Terminalia chevalieri* Diels, *Myrobalanus macroptera* (Guill. & Perr.) Kuntze) [17] is a tree up to 20 m in height. It is widespread in West Africa from Senegal to Cameroon, often on wet land [67].

### 5.1. Ethnopharmacology

Different parts of *T. macroptera* have been used for numerous ailments, including malaria [68,69,70], GI tract ailments (e.g., diarrhoea, gastritis, colitis, piles) [67,71,72], and infectious diseases including sexually transmitted diseases [73,74].

We carried out a systematic survey on the medicinal use of *T. macroptera* by traditional healers in three districts in Mali; Siby, Dioila and Dogonland [75]. Although there were regional differences, major areas of use were against pain and rheumatism (all areas), wounds (mainly in Siby), and hepatitis (mainly in Dogonland). Cough, diarrhoea and fever/malaria were also treated with *T. macroptera* preparations. Root bark, stem bark and leaves were used, but *Loranthus* parasitic plants on this tree were often employed in Dogonland.

### 5.2. Chemistry

The first published research on the constituents of *T. macroptera* appears to be by Prista et al. [76], reporting on the identification of chlorogenic acid and quercetin, two very common phenolic natural products. Other flavonoids isolated from *T. macroptera* flowers include the flavone *C*-glucosides orientin, isoorientin [77], vitexin, isovitexin [78,79] and the flavonol glycosides isorhamnetin 3-*O*-(6-*O*-α-l-rhamnosyl)-β-d-glucoside, quercetin 3-*O*-(6-*O*-β-d-glucosyl)-α-l-rhamnoside, quercetin 3-*O*-β-d-glucoside and quercetin 3-*O*-(6-*O*-α-l-rhamnosyl)-β-d-glucoside [80]. Unidentified proantho-cyanidins have been reported to occur in the leaf extract [81].

Other polyphenols have been found, as well. Conrad et al [82] reported a new phenolic glucoside, vanillic acid 4-*O*-β-d-(6´-*O*-galloyl) glucopyranoside from the bark, and Kone et al [79] found a series of benzoic and cinnamic acids in the stem and root bark extracts of the plant. Ellagitannins, some of them new natural products, have been reported [82,83,84]. Ellagic acid and methylellagic acids were reported from the heartwood in an early investigation [85].

The other major group of natural products reported from *T. macroptera* is the terpenoids, more specifically triterpenoids. Idemudia [85] reported the presence of terminolic acid; more recently, 23-galloylarjunolic acid and its glucosyl ester [86] and glucosides of 24-deoxysericoside and chebuloside II [78] have been identified.

Research on the polysaccharides of *T. macroptera* has recently been carried out in our department [87,88,89]. Pectins are important in this plant, and their biological activities have been discussed below (Biological activity, Section 5.3).

**Figure 4:**
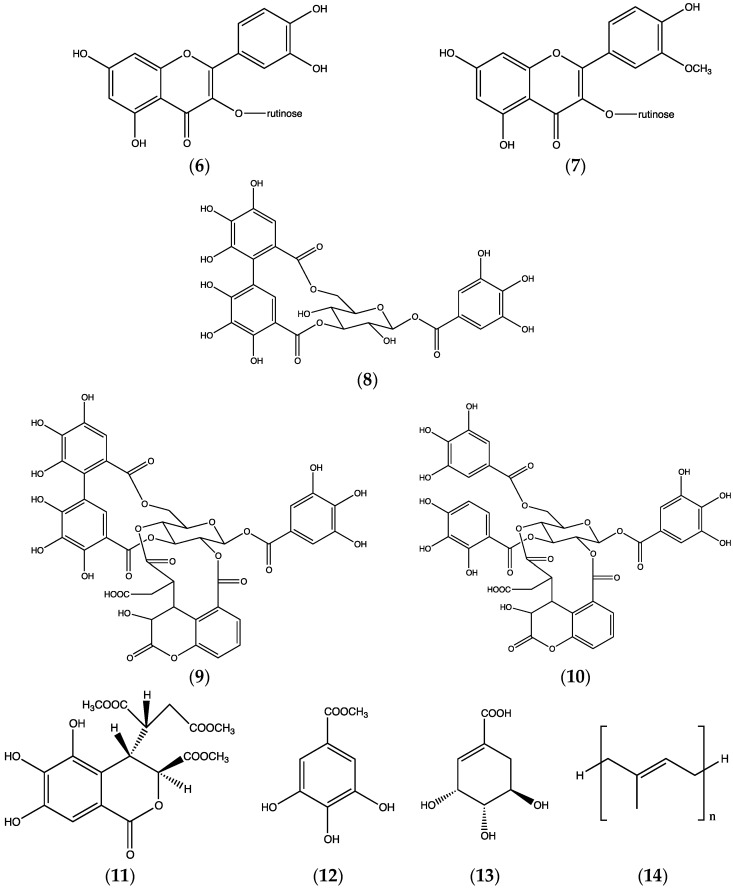
Constituents of *Terminalia macroptera* leaves: Flavonoids (**6**–**7**), ellagitannins (**8**–**10**), other constituents (**11**–**14**).

We investigated leaf constituents in *T. macroptera*, since this part of the plant was not well known. A series of compounds was isolated and identified [13] from the methanolic extract (Figure 4): The flavonoids rutin (**6**) and narcissin (**7**), the hydrolyzable tannins corilagin (**8**), chebulagic acid (**9**), chebulinic acid (**10**) and chebulic acid trimethyl ester (**11**), methyl gallate (**12**) and shikimic acid (**13**). In the dichloromethane extract, poly-*cis*-isoprene (**14**; calculated average chain length ca 25 units) was the main constituent. All of these compounds are new to *T. macroptera*, and chebulic acid trimethyl ester is a new compound. It may, however, be an artifact, formed from chebulic acid during methanol extraction.

### 5.3. Biological Activity

Pharmacological studies of *T. macroptera* were started in the 1990s by Silva and co-workers. In a series of papers [74,90,91,92,93], extracts of roots and leaves were investigated for antimicrobial activity towards a series of pathogenic microorganisms, including *Neisseria gonorrheae* and *Helicobacter pylori*. Other groups have investigated antimicrobial effects, as well: Antibacterial and/or antifungal effects were reported [81,82,94,95,96]. Effects on the malaria protozoa *Plasmodium falciparum* [68,97] as well as on another pathogenic protozoa, *Trypanosoma brucei* [97] might be of special importance in view of the serious nature of the diseases caused by these organisms.

The pharmacology of the pectic polysaccharides from *T. macroptera* was recently investigated [87,88,89]. These polysaccharides were found to have immunomodulatory and complement fixing properties. Interestingly, such activities were present in preparations made in the same way as traditional healers do [88].

We found [13] that the methanol crude extract had high radical scavenger activity (6.2 ± 0.4 μg/mL) and showed moderate inhibition (52 ± 5 μg/mL) of xanthine oxidase, an enzyme involved in production of superoxide radical anion. Xanthine oxidase inhibition is of medicinal importance in the treatment of gout. While poly-*cis*-isoprene was inactive in all our assays, the other isolated compounds showed various activities. Corilagin and chebulagic acid were very good radical scavengers, with IC_50_ values less than half of the positive control quercetin. Due to lack of material, chebulinic acid could not be tested.

Rutin and chebulagic acid inhibited xanthine oxidase. They were, however, less active than quercetin (positive control). Shikimic acid was inactive [13]. Toxicity towards brine shrimp was low (LD_50_ > 100 μg/mL for all extracts, >200 μM for all pure compounds) [14]. This test is commonly used as an indication of general toxicity [98]. The crude methanol extract had good activity as a 15-lipoxygenase inhibitor (IC_50_ 27.9 ± 1.5 μg/mL, comparable to the positive control quercetin) and an α-glucosidase inhibitor (IC_50_ 0.47 ± 0.03 μg/mL, much more active than the positive control acarbose with an IC_50_ value of 130 ± 18 μg/mL). In 15-lipoxygenase inhibition, both chebulagic acid, corilagin and narcissin were considerably more active than the positive control quercetin, while chebulagic acid showed remarkable inhibitory activity towards α-glucosidase (IC_50_ < 0.1 μM). Corilagin was less active, but being present in much higher concentration in the extract, it may well be the most important component in this respect [14,15]. In sum, we have found that *T. macroptera* leaves constitute a rich source of bioactive compounds, with good activity both as an antioxidant and radical scavenger and as an inhibitor of α-glucosidase. This might be of importance for their use by traditional healers.

Biological activities in vitro (radical scavenging; enzyme inhibition (α-glucosidase, 15-lipoxygenase, xanthine oxidase); complement fixation) and in vivo (toxicity towards brine shrimp) of ethanol and water extracts of root bark, stem bark and leaves of *T. macroptera* were investigated in a separate set of experiments [99]. Ethanol extracts of root bark and stem bark showed the highest activity. Radical scavenging and enzyme inhibition was correlated to total phenolic content, while complement fixation was not. The extracts were non-toxic towards brine shrimp.

The ethnopharmacological use of the plant against GI tract ailments might be related to its content of gallotannins and ellagitannins. The polysaccharides of the plant have anti-inflammatory properties in vitro, but no clinical studies have been carried out.

## 6. Conclusions

The plants *Biophytum umbraculum, Burkea africana, Lannea velutina* and *Terminalia macroptera* are used in traditional medicine in Mali against diverse ailments. Extracts of these plants show a variety of biological effects in vitro and in vivo. These effects may be related to the medicinal use of these plants, and may therefore indicate that their use in traditional medicine could have a rational basis. Clinical studies are, however, needed to draw conclusions on this.

## Figures and Tables

**Table 1:** Data for herbarium voucher samples of plants discussed in this review.

Plant	Deposited in	Registry Number	Article Reference
*Biophytum umbraculum*	DMT	2653	[10]
*Burkea africana*	DMT	no number	[11]
		(registered under plant name)	
*Lannea velutina*	DMT	1014	[12]
*Terminalia macroptera*	DMT	2468	[13,14]

DMT: Department of Traditional Medicine, Bamako, Mali.

## References

[1] Iwu M.M. (2014). Handbook of African Medicinal Plants.

[2] Petrovska B.B. (2012). Historical review of medicinal plants’ usage. Pharmacogn. Rev..

[3] Paulsen B.S., Ekeli H., Johnson Q., Norum K.R. (2012). South African Traditional Medicinal Plants from Kwazulu-Natal.

[4] Watt J.M., Breyer-Brandwijk M.G. (1962). The Medicinal and Poisonous Plants of Southern and Eastern Africa: Being an Account of Their Medicinal and Other Uses, Chemical Composition, Pharmacological Effects and Toxicology in Man and Animal.

[5] Burkill H.M. (1985–2000). The Useful Plants of West Tropical Africa.

[6] Kuete V. (2013). Medicinal Plant Research in Africa: Pharmacology and Chemistry.

[7] Neuwinger H.D. (2000). African Traditional Medicine.

[8] Van Wyk B.E., Van Oudtshoorn B., Gericke N. (2009). Medicinal Plants of South Africa.

[9] Universitetet I Oslo The Malian Medicinal Plant Project. http://www.mn.uio.no/farmasi/english/research/projects/maliplants/.

[10] Pham A.T., Nguyen C., Malterud K.E., Diallo D., Wangensteen H. (2013). Bioactive flavone-C-glycosides of the African medicinal plant *Biophytum umbraculum*. Molecules.

[11] Mathisen E., Diallo D., Andersen Ø.M., Malterud K.E. (2002). Antioxidants from the bark of *Burkea africana*, an African medicinal plant. Phytother. Res..

[12] Maiga A., Malterud K.E., Diallo D., Paulsen B.S. (2006). Antioxidant and 15-lipoxygenase inhibitory activities of the Malian medicinal plants *Diospyros abyssinica* (Hiern) F. White (Ebenaceae), *Lannea velutina* A. Rich. (Anacardiaceae) and *Crossopteryx febrifuga* (Afzel) Benth. (Rubiaceae). J. Ethnopharmacol..

[13] Pham A.T., Malterud K.E., Paulsen B.S., Diallo D., Wangensteen H. (2011). DPPH radical scavenging and xanthine oxidase inhibitory activity of *Terminalia macroptera* leaves. Nat. Prod. Commun..

[14] Pham A.T., Malterud K.E., Paulsen B.S., Diallo D., Wangensteen H. (2014). α-Glucosidase inhibition, 15-lipoxygenase inhibition, and brine shrimp toxicity of extracts and isolated compounds from *Terminalia macroptera* leaves. Pharm. Biol..

[15] Wangensteen H., Diallo D., Paulsen B.S. (2015). Medicinal plants from Mali: Chemistry and biology. J. Ethnopharmacol..

[16] Pham A.T. (2014). Chemical, Biological and Ethnopharmacological Studies of Two Malian Medicinal Plants: Terminalia Macroptera and Biophytum Umbraculum. Ph.D. Thesis.

[17] The Plant List. www.theplantlist.org.

[18] Diallo D., Sogn C., Samaké F.B., Paulsen B.S., Michaelsen T.E., Keita A. (2002). Wound healing plants in Mali, the Bamako region. An ethnobotanical survey and complement fixation of water extracts from selected plants. Pharm. Biol..

[19] Grønhaug T.E., Glæserud S., Skogsrud M., Ballo N., Bah S., Diallo D., Paulsen B.S. (2008). Ethnopharmacological survey of six medicinal plants from Mali, West-Africa. J. Ethnobiol. Ethnomed..

[20] Inngjerdingen K.T., Coulibaly A., Diallo D., Michaelsen T.E., Paulsen B.S. (2006). A complement fixing polysaccharide from *Biophytum petersianum* Klotzsch, a medicinal plant from Mali, West Africa. Biomacromolecules.

[21] Inngjerdingen M., Inngjerdingen K.T., Patel T.R., Allen S., Chen X., Rolstad B., Morris G.A., Harding S.E., Michaelsen T.E., Diallo D. (2008). Pectic polysaccharides from *Biophytum petersianum* Klotzsch, and their activation of macrophages and dendritic cells. Glycobiology.

[22] Grønhaug T.E., Kiyohara H., Sveaass A., Diallo D., Yamada H., Paulsen B.S. (2011). Beta-D-(1→4)-galactan-containing side chains in RG-I regions of pectic polysaccharides from *Biophytum petersianum* Klotzsch. contribute to expression of immunomodulating activity against intestinal Peyer’s patch cells and macrophages. Phytochemistry.

[23] Santoso B., Kilmaskossu A., Sambodo P. (2007). Effects of saponin from *Biophytum petersianum* Klotzsch on ruminal fermentation, microbial protein synthesis and nitrogen utilization in goats. Anim. Feed Sci. Technol..

[24] Hariadi B.T., Santoso B. (2010). Evaluation of tropical plants containing tannin on in vitro methanogenesis and fermentation parameters using rumen fluid. J. Sci. Food Agric..

[25] Bucar F., Jachak S.M., Kartnig T., Schubert-Zsilavecz M. (1998). Phenolic compounds from *Biophytum sensitivum*. Pharmazie.

[26] Hatano T., Mizuta S., Ito H., Yoshida T. (1999). C-glycosidic flavonoids from *Cassia occidentalis*. Phytochemistry.

[27] Heredia-Vieira S.C., Simonet A.M., Vilegas W., Macías F.A. (2015). Unusual C,O-fused glucosylapigenins from *Serjania marginata* leaves. J. Nat. Prod..

[28] Titrikou S., Aklikokou A.K., Gbeassor M. (1998). Effet de l’extrait de *Biophytum petersianum* (Oxalidaceae) Klotzsch sur le systeme cardiovasculaire de cobaye. Pharm. Med. Tradit. Afr..

[29] Titrikou S., Eklu-Gadegbeku K., Mouzou A., Aklikokou K., Gbeassor M. (2007). Calcium antagonistic activity of *Biophytum petersianum* on vascular smooth muscles of Wistar rats. Iran. J. Pharmacol. Ther..

[30] Kodjo K.M., Contesse V., Do Rego J.L., Aklikokou K., Titrikou S., Gbeassor M., Vaudry H. (2006). In vitro effects of crude extracts of *Parkia biglobosa* (Mimosaceae), *Stereospermum kunthianum* (Bignoniaceae) and *Biophytum petersianum* (Oxalidaceae) on corticosteroid secretion in rat. J. Steroid Biochem. Mol. Biol..

[31] Inngjerdingen K.T., Langerud B.K., Rasmussen H., Olsen T.K., Austarheim I., Grønhaug T.E., Aaberge I.S., Diallo D., Paulsen B.S., Michaelsen T.E. (2013). Pectic polysaccharides isolated from Malian medicinal plants protect against *Streptococcus pneumoniae* in a mouse pneumococcal pneumonia infection model. Scand. J. Immunol..

[32] Uchiumi F., Hatano T., Ito H., Yoshida T., Tanuma S.I. (2003). Transcriptional suppression of the HIV promoter by natural compounds. Antivir. Res..

[33] Willcox M., Sanogo R., Diakite C., Giani S., Paulsen B.S., Diallo D. (2012). Improved traditional medicines in Mali. J. Altern. Complement. Med..

[34] Austarheim I., Pham A.T., Nguyen C., Zou Y.F., Diallo D., Malterud K.E., Wangensteen H. (2016). Antiplasmodial, anti-complement and anti-inflammatory in vitro effects of *Biophytum umbraculum* Welw. traditionally used against cerebral malaria in Mali. J. Ethnopharmacol..

[35] Nonyane F., Masupa T. *Burkea africana* Hook. http://www.plantzafrica.com/plantab/burkeaafricana.htm.

[36] Delaveau P.A., Desvignes E., Adoux E., Tessier A.M. (1979). Baguettes frotte-dents d´ Afrique occidentale. Examen chimique et microbiologique. Ann. Pharm. Fr..

[37] Pedersen M.E., Vestergaard H.T., Hansen S.L., Bah S., Diallo D., Jäger A.K. (2009). Pharmacological screening of Malian medicinal plants used against epilepsy and convulsions. J. Ethnopharmacol..

[38] Maroyi A. *Burkea africana* Hook. http://uses.plantnet-project.org/en/Burkea_africana_(PROTA).

[39] Correira da Silva A., Paiva M.Q. (1971). Comparison of the pharmacodynamic activity of bark extracts from *Burkea africana* with one of its alkaloid constituents. An. Fac. Farm. Porto..

[40] Ferreira M.A. (1972). Indole alkaloids from *Burkea africana*. An. Fac. Farm. Porto..

[41] Ferreira M.A. (1973). Chemical study of *Burkea africana*. I. Identification of β-sitosterol and tetrahydroharman. Garcia de Orta. Ser. Farmacogn..

[42] Ferreira M.A. (1973). Indolic alkaloids from *Burkea africana*. II. Characterization of harmane and dihydroharmane. Garcia de Orta Ser. Farmacogn..

[43] Watson R., Fowden L. (1973). Amino acids of *Caesalpinia tinctoria* and some allied species. Phytochemistry.

[44] Malan J.C.S., Young D.A., Steenkamp J.A., Ferreira D. (1988). Oligomeric flavonoids. Part 2. The first profisetinidins with dihydroflavonol constituent units. J. Chem. Soc. Perkin Trans. 1.

[45] Bam M., Malan J.C.S., Young D.A., Brandt E.V., Ferreira D. (1990). Profisetinidin-type 4-arylflavan-3-ols and related δ-lactones. Phytochemistry.

[46] Davidson B.C. (1998). Seasonal changes in leaf lipid and fatty acid composition of nine plants consumed by two African herbivores. Lipids.

[47] Diallo D., Marston A., Terreaux C., Touré Y., Paulsen B.S., Hostettmann K. (2001). Screening of Malian medicinal plants for antifungal, larvicidal, molluscicidal, antioxidant and radical scavenging activities. Phytother. Res..

[48] Cordier W., Gulumian M., Cromarty A.D., Steenkamp V. (2013). Attenuation of oxidative stress in U937 cells by polyphenolic-rich bark fractions of *Burkea africana* and *Syzygium cordatum*. BMC Complement. Altern. Med..

[49] Neya B., Hakkou M., Pétrissans M., Gérardin P. (2004). On the durability of *Burkea africana* heartwood: Evidence of biocidal and hydrophobic properties responsible for durability. Ann. For. Sci..

[50] Steenkamp V., Fernandes A.C., Van Rensburg C.E.J. (2007). Screening of Venda medicinal plants for antifungal activity against *Candida albicans*. S. Afr. J. Bot..

[51] Tanko Y., Iliya B., Mohammed A., Mahdi M.A., Musa K.Y. (2011). Modulatory effect of ethanol stem bark extract of *Burkea africana* on castor oil induced diarrhoea on experimental animals. Arch. Appl. Sci. Res..

[52] Molander M., Nielsen L., Søgaard S., Staerk D., Rønsted N., Diallo D., Chifundera K.Z., Van Staden J., Jäger A.K. (2014). Hyaluronidase, phospholipase A_2_ and protease inhibitory activity of plants used in traditional treatment of snakebite-induced tissue necrosis in Mali, DR Congo and South Africa. J. Ethnopharmacol..

[53] Dzoyem J.P., Eloff J.N. (2015). Anti-inflammatory, anticholinesterase and antioxidant activity of leaf extract of twelve plants used traditionally to alleviate pain and inflammation in South Africa. J. Ethnopharmacol..

[54] Mathisen E. (1999). Radical Scavengers and Antioxidants from Burkea Africana, an African Medicinal Plant. Master’s Thesis.

[55] Rane M.M., Mengi S.A. (2003). Comparative effect of oral administration and topical application of alcoholic extract of *Terminalia arjuna* bark on incision and excision wounds in rats. Fitoterapia.

[56] Kisseih E., Lechtenberg M., Petereit F., Sendker J., Zacharski D., Brandt S., Agyare C., Hensel A. (2015). Phytochemical characterization and in vitro wound healing activity of leaf extracts from *Combretum mucronatum* Schum. & Thonn.: Oligomeric procyanidins as strong inductors of cellular differentiation. J. Ethnopharmacol..

[57] Taïta P. (2003). Use of woody plants by locals in Mare aux Hippopotames biosphere reserve in western Burkina Faso. Biodivers. Conserv..

[58] Belem B., Nacoulma B.M.I., Gbangou R., Kambou S., Hansen H.H., Gausset Q., Lund S., Raebild A., Lompo D., Ouedraogo M. (2007). Use of non wood forest products by local people bordering the “Parc national Kaboré Tambi”, Burkina Faso. J. Transdiscipl. Environ. Stud..

[59] Jansen P.C.M. *Lannea velutina* A. Rich. PROTA. http://uses.plantnet-project.org/en/Lannea_velutina_(PROTA).

[60] Maiga A., Diallo D., Fane S., Sanogo R., Paulsen B.S., Cisse B. (2005). A survey of toxic plants on the market in the district of Bamako, Mali: Traditional knowledge compared with a literature search of modern pharmacology and toxicology. J. Ethnopharmacol..

[61] Gueye M., Ayessou N.C., Koma S., Diop S., Akpo L.E., Samb P.I. (2014). Wild fruits traditionally gathered by the Malinke ethnic group in the edge of Niokolo Koba park (Senegal). Am. J. Plant Sci..

[62] Pageard R. (1967). Plantes à brûler chez les Bambara. J. Soc. Afr..

[63] Maiga A., Malterud K.E., Mathisen G.H., Paulsen R.E., Thomas-Oates J., Bergström E., Reubsaet L., Diallo D., Paulsen B.S. (2007). Cell protective antioxidants from the root bark of *Lannea velutina* A. Rich., a Malian medicinal plant. J. Med. Plants Res..

[64] Sérémé A., Millogo Rasolodimby J., Guinko S., Nacro M. (2008). Concentration en tanins des organes de plantes tannifères du Burkina Faso. J. Soc. Ouest.-Afr. Chim..

[65] Ouattara L., Koudou J., Zongo C., Barro N., Savadogo A., Bassole I.H.N., Ouattara A.S., Traore A.S. (2011). Antioxidant and antibacterial activities of three species of *Lannea* from Burkina Faso. J. Appl. Sci..

[66] Cires M.J., Wong X., Carrasco-Pozo C., Gotteland M. (2017). The gastrointestinal tract as a key target organ for the health-promoting effects of dietary proanthocyanidins. Front. Nutr..

[67] Arbonnier M. (2004). Trees, Shrubs and Lianas of West African Dry Zones.

[68] Sanon S., Ollivier E., Azas N., Mahiou V., Gasquet M., Ouattara C.T., Nebie I., Traore A.S., Esposito F., Balansard G. (2003). Ethnobotanical survey and in vitro antiplasmodial activity of plants used in traditional medicine in Burkina Faso. J. Ethnopharmacol..

[69] Benoit-Vical F., Soh P.N., Saléry M., Harguem L., Poupat C., Nongonierma R. (2008). Evaluation of Senegalese plants used in malaria treatment: Focus on *Chrozophora senegalensis*. J. Ethnopharmacol..

[70] Traore M.S., Baldé M.A., Diallo M.S.T., Baldé E.S., Diané S., Camara A., Diallo A., Balde A., Keïta A., Keita S.M. (2013). Ethnobotanical survey on medicinal plants used by Guinean traditional healers in the treatment of malaria. J. Ethnopharmacol..

[71] Etuk E.U., Uhwah M.O., Ajagbonna O.P., Onyeyili P.A. (2009). Ethnobotanical survey and preliminary evaluation of medicinal plants with antidiarrhoea properties in Sokoto state, Nigeria. J. Med. Plants Res..

[72] Kayode J., Ige O.E., Adetogo T.A., Igbakin A.P. (2009). Conservation and biodiversity erosion in Ondo state, Nigeria: (3). Survey of plant barks used in native pharmaceutical extraction in Akoko region. Ethnobot. Leafl..

[73] Kayode J., Jose R.A., Ige O.E. (2009). Conservation and biodiversity erosion in Ondo state, Nigeria: (4). Assessing botanicals used in the cure of sexually transmitted diseases in Owo region. Ethnobot. Leafl..

[74] Silva O., Ferreira E., Pato M.V., Canica M., Gomes E.T. (2002). In vitro anti-*Neisseria gonorrhoeae* activity of *Terminalia macroptera* leaves. FEMS Microbiol. Lett..

[75] Pham A.T., Dvergsnes C., Togola A., Wangensteen H., Diallo D., Paulsen B.S., Malterud K.E. (2011). *Terminalia macroptera*, its current medicinal use and future perspectives. J. Ethnopharmacol..

[76] Prista L.N., De Almeida e Silva L., Alves A.C. (1962). Phytochemical study of the barks and leaves of *Terminalia macroptera*. Garcia de Orta.

[77] Nongonierma R., Proliac A., Raynaud J. (1987). Two mono-*C*-glycosyl flavonoids from the flowers of *Terminalia macroptera* Guill. et Perr. (Combretaceae). Pharmazie.

[78] Nongonierma R., Proliac A., Raynaud J. (1988). Vitexin and isovitexin in the flowers of *Terminalia macroptera* Guill. et Perr. (Combretaceae). Pharmazie.

[79] Kone D., Diop B., Diallo D., Djilani A., Dicko A., Uddin J. (2012). Identification, quantitative determination, and antioxidant properties of polyphenols of some Malian medicinal plant parts used in folk medicine. Macro to Nano Spectroscopy.

[80] Nongonierma R., Proliac A., Raynaud J. (1990). *O*-glycosyl flavonoids of flowers from *Terminalia macroptera* Guill. et Perr. (Combretaceae). Pharm. Acta Helv..

[81] Karou S.D., Tchacondo T., Tchibozo M.A.D., Anani K., Ouattara L., Simpore J., De Souza C. (2012). Screening Togolese medicinal plants for few pharmacological properties. Pharmacogn. Res..

[82] Conrad J., Vogler B., Klaiber I., Reeb S., Guse J.H., Roos G., Kraus W. (2001). Vanillic acid 4-*O*-β-d-(6′-*O*-galloyl) glucopyranoside and other constituents from the bark of *Terminalia macroptera* Guill. et Perr. Nat. Prod. Lett..

[83] Silva O., Gomes E.T., Wolfender J.L., Marston A., Hostettmann K. (2000). Application of high performance liquid chromatography coupled with ultraviolet spectroscopy and electrospray mass spectrometry to the characterization of ellagitannins from *Terminalia macroptera* roots. Pharm. Res..

[84] Conrad J., Vogler B., Reeb S., Klaiber I., Papajewski S., Roos G., Vasquez E., Setzer M.C., Kraus W. (2001). Isoterchebulin and 4,6-*O*-isoterchebuloyl-d-glucose, novel hydrolyzable tannins from *Terminalia macroptera*. J. Nat. Prod..

[85] Idemudia O.G. (1970). Terpenoids of Nigerian *Terminalia* species. Phytochemistry.

[86] Conrad J., Vogler B., Klaiber I., Roos G., Walter U., Kraus W. (1998). Two triterpene esters from *Terminalia macroptera* bark. Phytochemistry.

[87] Zou Y.F., Zhang B.Z., Barsett H., Inngjerdingen K.T., Diallo D., Michaelsen T.E., Paulsen B.S. (2014). Complement fixing polysaccharides from *Terminalia macroptera* root bark, stem bark and leaves. Molecules.

[88] Zou Y.F., Zhang B.Z., Inngjerdingen K.T., Barsett H., Diallo D., Michaelsen T.E., Paulsen B.S. (2014). Complement activity of polysaccharides from three different plant parts of *Terminalia macroptera* extracted as healers do. J. Ethnopharmacol..

[89] Zou Y.F., Barsett H., Ho G.T.T., Inngjerdingen K.T., Diallo D., Michaelsen T.E., Paulsen B.S. (2015). Immunomodulating pectins from root bark, stem bark and leaves of the Malian medicinal plant *Terminalia macroptera*: Structure activity relations. Carbohydr. Res..

[90] Silva O., Duarte A., Cabrita J., Pimentel M., Diniz A., Gomes E. (1996). Antimicrobial activity of Guinea-Bissau traditional remedies. J. Ethnopharmacol..

[91] Silva O., Duarte A., Pimentel M., Viegas S., Barroso H., Machado J., Pires I., Cabrita J., Gomes E. (1997). Antimicrobial activity of *Terminalia macroptera* root. J. Ethnopharmacol..

[92] Silva O., Ferreira E., Vaz Pato M., Gomes E. (1997). Guinea-Bissau’s plants: In vitro susceptibility studies on *Neisseria gonorrheae*. Int. J. Pharmacogn..

[93] Silva O., Viegas S., De Mello-Sampayo C., Costa M.J.P., Serrano R., Cabrita J., Gomes E.T. (2012). Anti-*Helicobacter pylori* activity of *Terminalia macroptera* root. Fitoterapia.

[94] Batawila K., Kokou K., Koumaglo K., Gbéassor M., De Foucault B., Bouchet P., Akpagana K. (2005). Antifungal activities of five Combretaceae used in Togolese traditional medicine. Fitoterapia.

[95] Traoré M.S., Baldé M.A., Camara A., Baldé E.S., Diané S., Diallo M.S.T., Keita A., Cos P., Maes L., Pieters L. (2015). The malaria co-infection challenge: An investigation into the antimicrobial activity of selected Guinean medicinal plants. J. Ethnopharmacol..

[96] Traore Y., Ouattara K., Ouattara A., Méité S., Bagré I., Konan K.F., Coulibaly A., Nathalie K.G. (2015). Evaluation of the antistaphylococcic activity of *Terminalia macroptera* Guill et Perr (*Combretaceae*) stem bark extracts. Am. J. Biosci..

[97] Traore M.S., Diane S., Diallo M.S.T., Balde E.S., Balde M.A., Camara A., Diallo A., Keita A., Cos P., Maes L. (2014). In vitro antiprotozoal and cytotoxic activity of ethnopharmacologically selected Guinean plants. Planta Med..

[98] McLaughlin J.L., Hostettmann K. (1991). Crown gall tumours on potato discs and brine shrimp lethality: Two simple bioassays for higher plant screening and fractionation. Methods in Plant Biochemistry.

[99] Zou Y.F., Ho G.T.T., Malterud K.E., Le N.H.T., Inngjerdingen K.T., Barsett H., Diallo D., Michaelsen T.E., Paulsen B.S. (2014). Enzyme inhibition, antioxidant and immunomodulatory activities, and brine shrimp toxicity of extracts from the root bark, stem bark and leaves of *Terminalia macroptera*. J. Ethnopharmacol..

